# Biomarkers of lipid metabolism in patients with juvenile idiopathic arthritis: relationship with disease subtype and inflammatory activity

**DOI:** 10.1186/s12969-021-00538-w

**Published:** 2021-05-03

**Authors:** Wellington Douglas Rocha Rodrigues, Roseli Oselka Saccardo Sarni, Fernando Luiz Affonso Fonseca, Annelyse Araújo, Claudio Arnaldo Len, Maria Teresa Terreri

**Affiliations:** Department of Pediatrics, Universidade Federal de São Paulo, Rua Borges Lagoa, 802 – Vila Clementino, São Paulo, SP CEP: 04038-001 Brazil

**Keywords:** Juvenile idiopathic arthritis, Acute-phase proteins, Apolipoproteins, Cholesterol

## Abstract

**Background:**

To describe the biomarkers of lipid metabolism in children and adolescents with polyarticular and systemic JIA and to relate them to diseases subtypes, diseases activity markers, and nutritional status.

**Methods:**

A cross-sectional study including 62 JIA patients was performed. The following variables were evaluated: disease activity and medications used, body mass index, height for age (z-score), skin folds (bicipital, tricipital, subscapular and suprailiac), food intake based on three 24-h food recalls, lipid profile (total cholesterol (CT), low-density lipoprotein (LDL), high-density lipoprotein (HDL), triglycerides (TG) and non-HDL (N-HDLc), glycemia and insulin, erythrocyte sedimentation rate (ESR), ultrasensitive C-reactive protein (us-CRP) and apolipoproteins A-I and B (Apo A-I and Apo B).

**Results:**

Dyslipidemia was observed in 83.3% of the patients. Based on classical lipid profile, low HDL-c levels was the most frequently alteration observed. Inadequate levels of LDL-c, Apo B and NHDL-c were significantly more frequent in the systemic JIA subtype when compared to the polyarticular subtype (*p* = 0.017, 0.001 and 0.042 respectively). Patients on biological therapy had a better adequacy of Apo A-I concentrations. The ESR showed a negative correlation with Apo A-I level (*r* = − 0.25, *p* = 0.047).

**Conclusion:**

We concluded that dyslipidemia is common in patients with JIA, especially in systemic subtype. The systemic subtype and an elevated ESR were associated with lower concentrations of Apo A-I, suggesting the participation of the inflammatory process.

## Background

Juvenile idiopathic arthritis (JIA) is the most common chronic rheumatic disease in the pediatric population [1, 2]. JIA is defined as chronic inflammation in one or more joints for a minimum period of 6 weeks in children aged 16 years or younger [3].

Advances in the diagnosis and treatment of JIA over the last few decades have modified outcomes in children and adolescents, resulting in a decrease in mortality due to the better controlof disease activity and reduction of secondary infectious complications. However, this group of patients coexists chronically with the disease, and they present early negative outcomes, such as osteoporosis and cardiovascular diseases later on [4].

Studies have shown that adult patients with rheumatoid arthritis have an increased risk of atherosclerotic disease [5, 6]. In this group, the risk of cardiovascular diseases is 30 to 60% higher than that in the general population [7]. Long-term JIA adult patients in remission might have subclinical signs of inflammation and cardiovascular risk, showed by an increase in the levels of inflammatory cytokines, endothelial activation, and oxidative stress markers and adipokines, molecules involved in the alteration of the vascular system [8].

The identification of cardiovascular risk in the pediatric age group is a major challenge, especially in patients with chronic conditions such as JIA [9]. Measurements of the medial-intimal thickness (MIE) and biomarkers of lipid metabolism have been proposed as mechanisms for achieving this [10–13]. In adult patients with rheumatoid arthritis, changes in apolipoprotein B (Apo B) concentrations and the Apo B/apolipoprotein A-I (Apo A-I) ratio were compared to those in the classical lipid profile have been independently associated with cardiovascular risk [14].

To our knowledge, there are no studies in the literature on children and adolescents with JIA involving lipid biomarkers such as Apo A-I and Apo B that consider the disease onset subtypes.

Thus, we aimed to describe the biomarkers of lipid metabolism in children and adolescents with polyarticular and systemic JIA and relate them to disease subtypes, disease activity markers, and nutritional status.

## Patients and methods

In this cross-sectional study, at our pediatric rheumatology outpatient clinic, we included 68 consecutive children and adolescents of both genders who were age 5 to 19 years and who had polyarticular or systemic onset JIA according to the International League of Associations for Rheumatology (ILAR) [15].

Patients who did not want to participate or who had insufficient data on disease onset were excluded. Therefore, the sample included 62 patients.

This study was approved by the local ethics and research committee, and informed consent and assent were obtained from the caregivers or caregivers and patients.

Data on the demographic, clinical, and laboratory findings were obtained, and a nutritional, food intake, and family cardiovascular risk assessment was performed. A blood sample was collected on the same day.

Active disease was diagnosed according to the Wallace criteria, and the patients were classified as inactive, in remission on medication, in remission off medication, and active [16].

The nutritional assessment was performed based on the body mass index (BMI) and height for age (H/A), and patients were classified according to World Health Organization data. Waist circumference was classified as increased when it was higher than the 90th percentile, according to Freedman et al. [17]. Arm circumference, triceps, and subscapular skinfolds were assessed and classified according to Frisancho [18]. Pubertal stage assessment was performed according to Marshall & Tanner, considering breast development (M) for girls and genitalia (G) for boys.

The food intake assessment was performed through three 24-h food recalls [19]. To calculate the intake of macronutrients, total fat, cholesterol, monounsaturated, polyunsaturated, saturated and trans fats, fiber, and sodium, the Nutrition Support Program (Icalcdiet/Unifesp) was used, based on American and Brazilian references.

After a 12-h fast, blood was collected for analysis of the following lipid metabolism biomarkers: total cholesterol (TC), lipid fractions [very-low-density lipoprotein cholesterol (VLDL-c), LDL-c, high-density lipoprotein cholesterol (HDL-c)], triglycerides (TG), Apo A-I and Apo B, insulin, glucose, and ultra-sensitive C-reactive protein (us-CRP). Insulin and us-CRP were determined using chemiluminescence and immunoturbidimetric methods, respectively.

Apo A-I and Apo B were measured using enzyme-linked immunosorbent assay (ELISA) PRO kits for human Apo A-I and Apo B (Mabtech, Cincinnati, OH, USA) and also evaluated glycemia by enzymatic colorimetric assay and erythrocyte sedimentation rate (ESR).

The HOMA-IR (homeostasis model assessment of insulin resistance) value, classified as increased when > 3.16 [20]. To classify the lipid profile, the cutoff points proposed by the American Academy of Pediatrics were adopted [21]. Values of non-HDL (NHDL-c) were classified according to the Bogalusa Heart Study [22]. The ratios of TC/HDL-c, LDL-c/HDL-c, Apo B/Apo A-I, TG/HDL, and LDL-c/Apo B were also calculated.

For the qualitative variables, Pearson’s Chi-square association test or Fisher’s exact test was used, and for the quantitative variables, the Mann-Whitney test was used. We used the median as a measure of central tendency and the interquartile range (IQ) (25–75%) as a measure of dispersion. To evaluate the correlations between cardiovascular risk factors and HDL-c and Apo A-I, the Spearman correlation test was performed. The level of significance was set at *p* < 0.05.

## Results

Table 1 shows the demographic, clinical, and food intake data of children and adolescents with systemic and polyarticular JIA subtypes. The median BMI was 17.9 (16.1–21.0), three patients (4.8%) were underweight, and 13 (21%) were overweight. Regarding the other anthropometric data, the patients had medians of − 0.29 (− 1.1–0.5) for Z-score height/age, 65 cm (58–71 cm) for waist circumference, 0.43 (0.40–0.46) for waist/height, 22 cm (19–24.7 cm) for arm circumference, 13 mm (9–18 mm) for triceps skinfold, 9 mm (6–13 mm) for subscapular skinfold and a 20.8% (15.4–26.7%) percentage of body fat. Regarding food intake, the patients presented a median of 1933.6 kcal (1795–2177 kcal) for total energy intake. None of the patients presented with a carbohydrate intake higher than recommended, although 3 (4.9%) presented with a protein intake higher than the recommendation, and 18 (29%) presented with a lipid intake higher than the recommendation. The patients had medians of 24.4 g (20.9–30.9 g) for saturated fat and 4.2 g (2.2–6.4 g) for trans-fat; 47 (75.8%) presented a saturated fat intake higher than the recommendation, and 49 (79%) presented a trans-fat intake higher than the recommendation (data not showed in table).

**Table 1:** Demographic, clinical and food intake data of children and adolescents with polyarticular and systemic JIA subtypes

**Variables**	***N*** **= 62**	
Female N (%)	46 (74.2)	
Age, Mean ± SD	7.7 ± 4.3	
Socio-economic classification N(%)		
A	5 (8.0)	
B/C	54 (87.1)	
D/E	3 (4.8)	
Pubertal stage N(%)		
Pubertal	26 (41.9)	
Pre/Pos-pubertal	36 (58.1)	
Disease subtype N(%)		
JIA RF+	10 (16.1)	
JIA RF-	39 (62.9)	
Systemic	13 (21.0)	
Disease follow up time (years), Mean ± SD	5.0 ± 3.4	
Disease activity N(%)		
Active	21 (33.9)	
Inactive	5 (8.0)	
Remission on medication	29 (46.8)	
Remission off medication	7 (11.3)	
Biological DMARDs N(%)	16 (25.8)	
Synthetic DMARDs N(%)	40 (64.5)	
Corticosteroids N(%)	6 (9.7)	
Cumulative dosis of corticosteroids (mg)	2620	1762.5–7483.7
CHAQ altered N(%)	17(27.4)	
CHAQ, Mean ± SD	1.2 (0.8)	

*CHAQ* Childhood Health Assessment Questionnaire, *JIA* Juvenile idiopathic arthritis, *DMARDs* Disease modified antirrheumatic drugs

Table 2 shows body mass index, disease activity, and laboratory and lipid profile findings in children and adolescents with juvenile idiopathic arthritis according to HDL-c level. There was no statistical difference in BMI between both groups (HDL adequate vs borderline-low) and in BMI between both groups (TG adequate vs borderline-high).

**Table 2:** Body mass index, disease activity and laboratory and lipid profile findings in children and adolescents with juvenile idiopathic arthritis according to HDL-c level

**Variables**	**HDL-c**		
	**Adequate** ***n*** **= 42**	**Borderline-low** ***n*** **= 20**	***P***
Pubescent, N (%)	33 (78.6)	9 (45)	0.559
BMI	17.9 (17–21.2)	18.6 (16.1–20.3)	0.952
Z-Score BMI/age > 1	7 (16.7)	6 (30)	0.228
CHAQ	0.27 (0.1–0.6)	0.55 (0.3–1.03)	0.292
> 0, N (%)	10 (23.8)	7 (35)	0.356
Disease activity			
Active, N (%)	16 (38.1)	5 (25)	0.308
us-CRP	1.2 (0.5–4.9)	2.8 (0.9–15)	0.312
≥ 3	14 (33.3)	10 (50.0)	0.208
ESR	6.5 (5.0–30.0)	19.5 (6.0–32.0)	0.191
≥ 20	15 (35.7)	10 (50.0)	0.407
HDL-c	50.0 (45.0–59.0)	46.0 (42.0–49.0)	0.271
Borderline/Low N (%)	14 (28.6)	6 (46.2)	0.351
LDL-c	86.4 (72.2–103.0)	84.4 (71.2–99.4)	0.646
Borderline/High	8 (19.0)	4 (20.0)	1.000
Triglycerides	66.5 (51.0–85.0)	62.0 (51.5–88.5)	0.821
Borderline/High	12 (28.6)	5 (25.0)	1.000
Apo A-1	118.0 (108.0–137.0)	100.0 (92.0–106.5)	**< 0.001**
Borderline/Low	23 (54.8)	19 (95.0)	**0.001**
Apo B	72.5 (58.0–84.0)	68.0 (58.5–83.5)	0.821
Borderline/Low	5 (11.9)	4 (20.0)	0.453
NHDL-c	99.0 (87.0–124.0)	96.5 (82.5–119.5)	0.474
Borderline/Low	12 (28.6)	5 (25.0)	1.000

Tests: Fisher’s exact or Chi-square test, or Pearson or Mann-Whitney, *p* < 0.05; median (IQ: 25–75)

*BMI* Body mass index, *CHAQ* Childhood Health Assessment Questionnaire, *us-CRP* Ultra-sensitive C reactive protein, *ESR* Erythrocyte sedimentation rate, *LDL-c* Low-density lipoprotein cholesterol, *HDL-c* High-density lipoprotein cholesterol, *NHDL* Non-high-density lipoprotein cholesterol, *Apo A-I* Apolipoprotein A-1, *Apo B* Apolipoprotein B

Dyslipidemia was observed in 83.3% of the patients when altered lipid biomarkers (CT, LDL-c, HDL-c, TG, NHDL-c, Apo A-I, and Apo B) were considered. Borderline and low levels of HDL was the most frequent dyslipidemia based on classical lipid profile (20 patients - 32.3%). A decrease in Apo A-I level was present in 42 patients (67.7%) and more frequent in patients who did not use biological agents (83% versus 16%). The frequency of borderline /high values of LDL-c was statistically higher in systemic JIA than in polyarticular JIA (*p* = 0.017). In addition, we observed a higher frequency of Apo B and NHDL-c alterations and higher values of Apo B/Apo A-1 ratio, CT/HDL-c ratio, and LDL-c/HDL-c ratio in systemic JIA. Regarding the HDL-c variable, we observed that patients with borderline /low lipoprotein had lower levels and a higher frequency of inadequate Apo A-I. We also observed that familial cardiovascular risk was present in 42 patients (67%).

We did not find an association of inadequate Apo A-I or HDL-c with the time of disease duration and disease subtype, use of glucocorticoid, and with score-z BMI. The association of Apo A-I with disease activity showed a value of *p* = 0.055. We also observed that patients off biological agents had a higher frequency of inadequate Apo A-I than patients on biological agents (76% vs. 43.8%, respectively) (*p* = 0.017). The Apo-A1 levels were not associated with laboratory findings, classic lipid profile, Apo B, or glucose levels.

The frequency of borderline/high LDL-c values was statistically higher in systemic JIA than in polyarticular JIA (*p* = 0.017). In addition, we also observed statistically higher values of Apo B, Apo B / Apo A-1 ratio, non-HDL-c, CT / HDL-c ratio, and LDL-c / HDL-c in systemic JIA (*p* = 0.03, 0.042, 0.043, and 0.037, respectively).

**Fig. 1:**
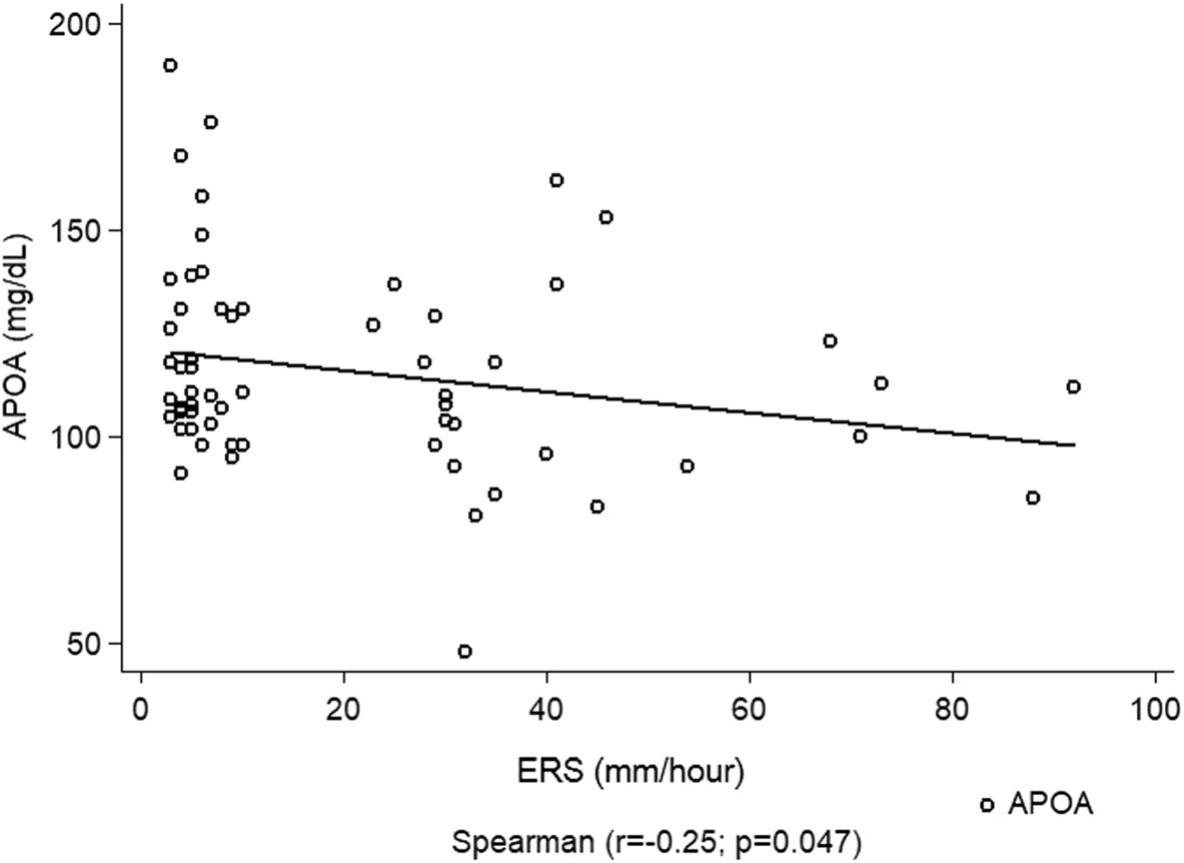
Correlation between levels of erythrocyte sedimentation rate and Apolipoprotein A-I in children and adolescents with juvenile idiopathic arthritis

We did not observe a correlation between CRP and the variables related to the lipid profile (Spearman’s correlation analysis). However, the ESR showed a negative correlation with Apo A-I levels (*r* = − 0.25, *p* = 0.047) (Fig. 1).

We also observed a positive correlation between the cumulative glucocorticoid dose and TC (*r* = 0.58, *p* = 0.018), LDL-c (*r* = 0.64, *p* = 0.008) and NHDL-c (*r* = 0.64; *p* = 0.007).

## Discussion

The present study showed that dyslipidemia affected more than three-quarters of JIA patients, with the predominance of borderline-low HDL-c based on classical lipid profile. Similar results were found in a retrospective study published by our research group [23]. We observed a higher frequency of increased LDL-c, NHDL-c, and Apo B in the systemic subtype than the polyarticular subtype. Systemic-onset JIA and elevated ESR were associated with lower concentrations of Apo A-I, suggesting the involvement, among other factors, of the inflammatory process.

We did not include oligoarticular JIA due to the absence of systemic activity and due to the fact that this JIA subtype rarely needs corticosteroids (except in the presence of uveitis).

Studies evaluating non-classical biochemical markers related to lipid metabolism in children and adolescents with autoimmune rheumatic diseases are scarce [12, 22, 24–26].

The mechanisms involved in the pathogenesis of dyslipidemia and cardiovascular risk in patients with JIA have not yet been fully elucidated. Inflammation with endothelial dysfunction and increased levels of proinflammatory cytokines have been described [27]. In addition, inadequate food intake, physical inactivity, overweight, and the use of certain medications, such as glucocorticoids, also aggravate dyslipidemia [28]. The use of glucocorticoids seems to play an important role in dyslipidemia, considering that, in our study, we observed an association between a higher cumulative dose and alterations in TC, LDL-c, and NHDL-c.

In patients with rheumatoid arthritis, the prevalence of dyslipidemia varies between 55 and 65% [29]. In our sample, the prevalence was 82.3% when we considered changes in Apo A-I and Apo B. Although these apolipoproteins are considered good biomarkers for cardiovascular risk, several studies did not consider them in the evaluation of dyslipidemia [24, 30, 31].

Anthropometric markers of adiposity, such as BMI, waist circumference, and intake of fats and carbohydrates, were normal for most patients. This fact could be explained by the role of the multi-professional team in our service and suggests important participation of the disease and its treatment in the pathogenesis of dyslipidemia.

Although glucocorticoids were used in only about 10% of our patients (two patients with systemic JIA and 4 patients with polyarticular JIA), we observed an association between the cumulative dose and dyslipidemia. Marangoni et al. reported a trend of association between glucocorticoid use and increased levels of LDL-c [30]. They also pointed out that the decrease in HDL-c level was not associated with the use of glucocorticoid medication The progressively increasing use of synthetic DMARDs and biological agents minimizes the harmful effects of glucocorticoids on lipid metabolism [32].

We know that systemic-onset JIA is associated with greater laboratory and inflammatory alterations, such as anemia, increased platelet count, and acute-phase proteins, and frequent use of corticosteroids. This explains the major changes in LDL-c, Apo B, and NHDL-c found in our patients with this JIA subtype. In a longitudinal study, Yeh et al. evaluated the lipid profile and atherogenic index of JIA patients after treatment with etanercept and found higher concentrations of HDL-c and lower TG and TC/HDL in the group they called responders (with the inactive disease) compared to non-responders [33]. The non-responder group, which consisted of patients with the systemic subtype, did not show improvement in the lipid profile. Other studies addressing dyslipidemia in JIA patients did not individualize the different subtypes and did not evaluate biomarkers such as Apo A-I and Apo B [24, 31].

Although an adequate HDL-c level was not associated with demographic and clinical characteristics, nutritional status, or food intake, patients with an adequate HDL-c had a higher value and higher frequency of normal levels of Apo A-I. In the literature, it is well-established that adequate levels of HDL-c, due to its antioxidant (mainly by the presence of Apo A-I), anti-inflammatory, antiatherogenic, antithrombotic functions, and cholesterol transportation, are related to a reduction in cardiovascular risk [34, 35]. However, it is worth mentioning that, in the presence of systemic inflammation, there may be a conversion of protective HDL-c to proinflammatory HDL-c, and the reduction in the production of Apo A-I is one of the proposed mechanisms of this transformation [34, 35].

Studies have shown an association between disease activity and dyslipidemia [33, 36]. Although we have a larger sample compared to other studies [33, 36], we found only a tendency towards an association between JIA activity and altered levels of Apo A-I; that is, patients with active disease had a higher frequency of reduction in Apo A-I than patients with inactive disease (in remission or not).

The decrease in the Apo A-I level was more frequent in patients who did not use biological agents (83% versus 16%). This can be explained by the action of these agents inhibiting the production of cytokines and, consequently, inflammation, thus improving the lipid profile.

Rodriguez-Jimenez showed that tumor necrosis factor-α (TNF-α) is associated with increased cardiovascular risk, increased hepatic CRP synthesis, and decreased HDL-c levels [37]. Under normal conditions, the major protein fraction of HDL is Apo A-I. However, the literature has shown that, in the presence of inflammation, mainly in increased interleukin-1 and TNF-α conditions, there is an increase in the production of serum amyloid A (SAA) by hepatocytes [38, 39]. This protein, on the other hand, has atherogenic action, when released into the bloodstream rapidly, it associates with the third fraction of HDL (HDL3), decreasing serum concentrations of Apo A-I. A study in patients with JIA has shown that anti-TNF-α therapy alters the proatherogenic lipid profile of these patients [40].

Interestingly, a negative correlation between the ESR and Apo A-I concentration was observed. Similar results were showed by Bakkaloglu et al. [41], that evaluated the lipid biomarkers of 37 patients with JIA, and found negative correlations between ESR and CRP with APO A-I. Although other inflammatory markers, such as proinflammatory cytokines, have been described as being present in active JIA [42], studies show that a high ESR is a good parameter of disease activity [43], when it is normal, it is one of the variables included in Wallace’s inactivity criteria [15]. Based on this, our findings suggest that, in the presence of high disease activity and/or a high ESR, the investigation of lipid metabolism biomarkers should be expanded.

This study has some limitations, such as the absence of a control group and the lack of an appropriate tool for the evaluation of the practice of physical activity by the patients. In addition, the sample size may have been insufficient to show significant differences between the various variables. However, this study is relevant and original because it is the first study on dyslipidemia, showing differences between the JIA subtypes.

## Conclusion

We concluded that dyslipidemia is frequently presented in patients with JIA, although the majority of patients presented a satisfactory nutritional status and absence of some components of metabolic syndrome. However, inadequate consumption of atherogenic lipids occurred frequently. Another important point was the association between the presence of systemic JIA and an elevated ESR with a higher frequency of an altered lipid profile. We also observed that the use of biological agents might be a protective factor for dyslipidemia. There was no association of BMI and HDL-c levels.

Therefore, future studies should involve, in addition to lipid biomarkers such as Apo A-I and Apo B, the evaluation of biomarkers of inflammation (cytokines), direct markers of vascular injury, and the performance of imaging methods to measure atherosclerotic risk to understand the cardiovascular risk in these patients and thus be able to outline strategies and interventions to reduce this risk.

## Data Availability

The datasets generated during and/or analyzed during the current study are not publicly available due [REASON WHY DATA ARE NOT PUBLIC]. Still, they are available from the corresponding author on a reasonable request.

## Abbreviations

JIA: Juvenile idiopathic arthritis
MIE: Medial-intimal thickness
Apo A-I: Apolipoprotein A-I
Apo B: Apolipoprotein B
lLAR: International League of Associations for Rheumatology
BMI: Body mass index
H\A: Height for age
UNIFESP: Universidade Federal de São Paulo
TC: Total cholesterol
VLDL: Lipid fractions [very-low-density lipoprotein cholesterol
LDL-c: Low-density lipoprotein cholesterol
HDL-c: High-density lipoprotein cholesterol
TG: Triglycerides
us-CRP: Ultra-sensitive C-reactive protein (us-CRP)
ESR: Erythrocyte sedimentation rate
HOMA-IR: Homeostasis model assessment of insulin resistance
NHDL-c: Non-high-density lipoprotein cholesterol
IQ: Interquartile range
DMARD: Disease-modifying antirheumatic drugs
TNF-α: Tumor necrosis factor-α
SAA: Serum amyloid A
